# Content comparison of haemophilia specific patient-rated outcome measures with the international classification of functioning, disability and health (ICF, ICF-CY)

**DOI:** 10.1186/1477-7525-8-139

**Published:** 2010-11-25

**Authors:** Silvia Riva, Monika Bullinger, Edda Amann, Sylvia von Mackensen

**Affiliations:** 1 Institute of Medical Psychology, Centre of Psychosocial Medicine, University Medical Centre Hamburg-Eppendorf, Germany; 2 Department of Medicine and Medical Sciences, A. Bianchi Bonomi Haemophilia and Thrombosis Centre, University of Milan and IRCCS Ca' Granda Foundation, Maggiore Hospital, Milan, Italy; 3 Department of Statistical Medicine and Informatics, University of Medicine, Innsbruck, Austria

## Abstract

**Background:**

Patient-Reported Outcomes (PROs) are considered important outcomes because they reflect the patient's experience in clinical trials. PROs have been included in the field of haemophilia only recently.

**Purpose:**

Comparing the contents of PROs measures used in haemophilia, based on the ICF/ICF-CY as frame of reference.

**Methods:**

Haemophilia-specific PROs for adults and children were selected on the grounds of international accessibility. The content of the selected instruments were examined by linking the concepts within the items of these instruments to the ICF/ICF-CY.

**Results:**

Within the 5 selected instruments 365 concepts were identified, of which 283 concepts were linked to the ICF/ICF CY and mapped into 70 different categories. The most frequently used categories were "b152: Emotional functions" and "e1101: Drugs".

**Conclusions:**

The present paper provides an overview on current PROs in haemophilia and facilitates the selection of appropriate instruments for specific purposes in clinical and research settings. This work was made possible by the grant of the European Murinet Project (Multidisciplinary Research Network on Health and Disability in Europe).

## Introduction

### Haemophilia

Haemophilia is a rare inherited X-linked coagulation disorder caused by deficiencies of the clotting factor VIII (FVIII: haemophilia A) or of factor IX (FIX: haemophilia B). The prevalent haemophilia is haemophilia A (1 out of 10,000 inhabitants) and for haemophilia B (1 out 30,000 inhabitants). Haemophilia A and B are the most frequent clinically severe inherited bleeding disorders [1,2]. According to factor activity levels, haemophilia is classified as: severe (<1%), moderate (1-5%) or mild (6-25%) [2].

The clinical hallmark of haemophilia is recurrent spontaneous bleeding, most frequently in joints such as: ankles, elbows and knees as well as in muscles [3,4]. The treatment of haemophilia is based on the replacement of the missing clotting factor when bleeding occurs (on-demand treatment) or is made on a regular and continuous way regularly and continuously (prophylactic treatment) [5]. In the Western World prophylactic treatment in young haemophilic patients is considered the golden standard [6], while for adults the benefits of prophylaxis are still discussed [7,8]. Haemorrhages lead to a progressive worsening of the status of joints and muscles, thus impacting on patients' well-being and daily life activities [9-11]. Moreover, haemophilia is quite expensive, on average € 15,000 per patient monthly, which can increase dramatically when inhibitors occur [12]. In a period of increasing costs, more attention is given not only to clinical efficacy but also to patients' well-being. It becomes hence essential to clinically monitor each individual patient as well as patient groups and also to assess the patient's perspective via patient-reported outcomes (PROs) [13,14].

For an adequate assessment of PROs, standardized and validated instruments are necessary [14]. In order to recruit adequate sample sizes, studies in haemophilia are conducted mainly on an international level, therefore PRO instruments are needed to be developed and linguistically validated in more than one country.

### Patient-rated Outcomes (PROs)

The assessment of PROs is increasingly becoming more important in clinical trials as primary or secondary endpoints [15]. PROs elicit the direct patient report which allows the evaluation of the impact of a disease and its treatment on patients' well-being and functioning [16]. Examples of PROs include health status, health-related quality of life (HRQoL), treatment satisfaction (TS), level of functioning (FUN) and patient preferences/utilities (PP). PROs are used to assess the value of treatments from a patients' perspective and to recommend strategies for improved care. This is particularly important in chronic health conditions with high demands on it's care, such as haemophilia. In the past eight years, several haemophilia-specific PROs have been developed for children and adults [17], even though some of them have not been published in peer-reviewed journals yet, but they have already been included in several clinical trials.

*Health-related quality of life (HRQoL) *is considered the most important outcome among the PROs referring to patients' perception of well-being and functioning [18,19]. Several haemophilia-specific HRQoL instruments have recently been developed both for children, such as the European Haemo-QoL [20 ,21], the Canadian CHO-KLAT [22] and the American Quality of Life Instrument for young children with haemophilia [23]. Haemophilia-specific instruments for adults have been developed only recently such as the Hemofilia-QoL [24], the Medtap questionnaire [25] and the Haem-A-QoL questionnaire [26,27].

T*reatment satisfaction (TS) *has recently become a focus of interest, representing the individual rating of the process and outcomes of patients' treatment experience and is related to adherence and willingness to continue treatment [28,29]. A first haemophilia-specific treatment satisfaction questionnaire (Hemo-Sat) for adults and parents has been developed [30,27].

Finally, haemophilia-specific *assessments of the level of functioning (FUN) *have been developed for the subjective evaluation of patients' daily activities or functioning such as the haemophilia activity list (HAL) [31] or the HEP-Test-Q [32].

Since the selection of an appropriate instrument for a particular aim/goal is essential for planning any data collection, it is particularly interesting to examine the content of different PROs and to compare the concepts covered for a well-founded choice of instruments [10,14]. Such a comparative assessment of content comparison should be based on a universally accepted, well-defined, and standardized system of reference [33], which allows a detailed exploration and comparison of all the measures' contents for the choice of instruments in the field of haemophilia.

In addition to the International Classification of Diseases (ICD-10) [34], the World Health Organisation (WHO) emphasizes the importance of disease consequences thanks to the International Classification of Functioning, Disability and Health (ICF) and its version for children and youth (ICF-CY), independent from a specific disease [35,39].

### The ICF Framework

The dominant theoretical models of health outcomes or the consequences of disease have been the models developed by the World Health Organization [36]. The most recent version, the ICF is based on a biopsychosocial model integrating medical and social models.

**Figure 1:**
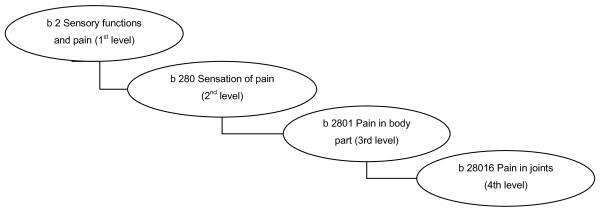
**Examples for different levels of the category 'body functions' of the ICF/ICF-CY**.

The ICF provides a model of functioning and disability that extends beyond disease and conceptualizes functioning in terms of 'body function', 'body structure', 'activity and participation', taking into account as contextual factors the so-called 'environmental' and 'personal factors'; the latter are not classified in the ICF because of the large social and cultural variance associated with them. The units of the ICF classification are called categories; they are organized within a hierarchical structure and are denoted by unique alphanumeric codes. Within each of the four major components ('Body Functions', 'Body Structures', 'Activities and Participation', 'Environmental Factors'), the categories are organized in an ordered system. Each component consists of chapters (categories at the first level), each chapter consists of second level categories, and in turn they are made up of categories at the third level, and so on. The ICF contains in total 1,454 categories, while the ICF-CY contains 1,685 categories. Figure 1 illustrates the structure of the ICF [35].

An important priority within the framework was the extension of the ICF taxonomy to children [37,38]. The recently published ICF-CY is a specific tool for children and adolescents [39] which takes into account three relevant issues: a) all the different components of childhood disability, b) the purpose of measurement in childhood, and c) the mediating roles of developmental and environmental factors on childhood disability [40]. The ICF-CY has been developed to be structurally consistent with the ICF for adults. A major difference between the ICF-CY and ICF is that the generic qualifiers from the adult ICF now include developmental aspects for children and young people in the ICF-CY. Descriptions of codes in the ICF-CY have been revised and expanded and new content was added to previously non-used codes. Codes were added to document characteristics as: adaptability, responsivity, predictability, persistence, and approachability. "Sensing" and "exploration of objects" codes were expanded as far as to include the "importance of learning" [41]. Since a child's main occupation is playing, it is also important to include more codes in this area.

### Why using the ICF in the health outcomes context?

The ICF/ICF-CY framework can be used as an orientation in choosing PRO instruments and in interpreting and comparing the results of different studies [33]. Within the many PRO instruments available, a common reference framework for functioning is of utmost importance and might improve outcome research [33].

The ICF can facilitate the selection of the most appropriate questionnaires for study intervention or clinical evaluation. Different publications have presented the results of the linking process between the most widely used condition-specific measures to the ICF [41,33,43].

The aim of this paper was to link haemophilia-specific PRO instruments to the ICF/ICF-CY in order to classify the contents of these PRO instruments with ICF/ICF-CY categories and to provide researchers with a tool that can help them in selecting the most appropriate instruments depending on their research question. This connection was done by examining how concepts inherent to cross-culturally validated PRO instruments in haemophilia are represented in the ICF (adults) or in the ICF-CY (children and adolescents).

This exercise was performed in the frame of the European Murinet project (Multidisciplinary Research Network on Health and Disability in Europe, MRTN-CT-2006-035794) [44]. In the Murinet project, disease-specific questionnaires were linked to the ICF and the ICF-CY. This represents an experimental and practical exercise converging with the aim of the Murinet project, which is intended to promote a European research activity in health and disability research and management and which is able to integrate several skills within the framework of the ICF classifications model.

## Methods

For the purpose of this research, a systematic literature review was performed in order to identify and select current haemophilia-specific PRO measures. The selection of instruments was related to self-rating modus, cross-cultural availability and accessibility of the instruments to the authors. The selected instruments are the sole translated and linguistically validated in more than two European languages. Paediatric and adult instruments were included in this selection in order to provide information about different age groups, which is important since haemophilia is quite a rare disease wherein patients from one age group are often not sufficient to be included in clinical trials.

In this linkage process the following disease-specific questionnaires were linked to the ICF and the ICF-CY (see table 1).

**Table 1:** Overview of selected PRO instruments.

Age Group	Type of Measures*	Name of questionnaire	Instrument	Dimension/subscales	N.°of Items**	Way of administration	Reliability	Validity in Haemophilia Examined	N° of languages	Use in haemophilia Research
**Children**	HRQoL	Haemo-QoL	Haemophilia -specific QoL Questionnaire for children patients and parents	8-12 (physical health, feeling, view of yourself, family, friends, perceived support, others, sports and school, dealing with haemophilia, treatment, future, relationships, global health)	I: 21 for children aged 4-7 II: 64 for children aged 8-12 III: 77 for adolescents aged 13-16	Self/Proxy	α = 0.85-0.91 (for the different age group versions)	yes	40	Epidemiological study (describing the quality of life of patients with reference to other chronic conditions), Clinical trials (to evaluate the potential benefits of different treatment regimes), Quality assurance (identifying the quality of care perceived by patients, e.g., in Haemophilia Comprehensive Care Centres (HCCC), Health-economic studies (assessing costs and benefits of haemophilia treatment with regard to economic indicators) and Routine treatment
										
**Adults**		Haem-A-QoL	Disease-specific questionnaire for adults patients	10 (physical health, feelings, view of yourself, sport and leisure, work and school, dealing with haemophilia, treatment, future, family planning, partnership and sexuality)	46	Self	α = 0.74-0.88	yes	32	
		Medtap (Haemo-QoL-A)	Haemophilia-specific QoL questionnaire for adults	4 (day-to-day activities, mood and feelings, work or school life, family life -social life, haemophilia treatment)	41	Self	α = 0.75-0.95	yes	20	
										
	TS	Hemo-Sat_A_	Haemophilia Treatment satisfaction Question- naire	6 (Ease and convenience, Efficacy Burden, Specialist/nurses, Centre/Hospital, General satisfaction with your treatment	34	Self	α = 0.71-0.95	yes	24	To evaluate patients' therapy and experience, in follow-up analysis
										
	Functioning	HEP-Test Q	Subjective assessment questionnaire of the effects of physical functioning in adult with haemophilia	5 (physical status, mobility strength-coordination, endurance, body perception)	25 + 1	Self	α = 0.70-0.90	yes	3	Evaluation of patients' daily activities or functioning, Follow-up analysis, Rehabilitation Programs

Three health-related Quality of Life instruments, one treatment satisfaction questionnaire and one questionnaire for physical functioning were included in the linking process.

### Health-related Quality of Life

The **Haemo-QoL **is the first haemophilia-specific HRQOL questionnaire and it is available in three age-group versions as self reports for children: version I for children aged 4-7 years old (21 items) as an interview, version II for children aged 8-12 years old (64 items) and version III for adolescents aged 13-16 years old (77 items), as well as three proxy versions for parents report respectively [21]. The initial development used parent's assessment of children's' quality of life as well as expert consensus of haemophilia treaters on relevant dimensions and items. On these grounds, an instrument for children has been constructed consisting of 8 to 12 dimensions according to different age groups, with fewer items for younger children in the domains 'physical health', 'feeling', 'view', 'family', 'friends', 'others', 'sport and school' and 'treatment'. Age groups II and III have in addition the domains 'perceived support' and 'dealing with haemophilia' and for adolescents the domains 'future' and 'relationship' are added. The three age group versions of the Haemo-QoL had acceptable internal consistency (ranging for the total score from α = 0.85-0.91 for the different age group versions) and retest reliability values for age groups II and III (ranging from r = 0.90-0.92), as well as possessed sufficient discriminant and convergent validity. The Haemo-QoL was originally validated in six European countries (Germany, Italy, France, Spain, Netherlands, and the UK) and it is now available in 40 languages, from which 32 are linguistically validated.

The **Medtap (Haemo-QoL-A) **is a HRQoL questionnaire specifically designed for adult haemophilia patients [25]. It consists of 41 items pertaining to 4 dimensions ('day-to-day activities', 'mood and feelings', 'work or school life, family and social life', 'haemophilia treatment'). The questionnaire asks how haemophilia and its treatment affect the life of patients in each dimension. The questionnaire was originally developed in the US, Germany and Spain. Initially, focus groups with haemophilia patients were conducted simultaneously in these countries to derive items. It shows quite satisfactory psychometric characteristics in terms of reliability (Cronbach's α = 0.75-0.95) and validity (convergent: correlation with life satisfaction scale; discriminant: differences for clinical subgroups concerning severity and target joints). The questionnaire is linguistically validated in several languages.

The **Haem-A-QoL **is a HRQoL questionnaire specifically designed for adult patients with haemophilia [26]. Item generation was derived from patient-based focus groups (n = 32) and expert groups organized with physicians and nurses in Italy. It was validated in 233 Italian adult patients [45] and consisted of 46 items pertaining to 10 dimensions ('physical health', 'feelings', 'view of yourself', 'sport and leisure', 'work and school', 'dealing with haemophilia', 'treatment', 'future', 'family planning', 'partnership and sexuality') and a total score. The psychometric characteristics showed quite good reliability values ranging from α = 0.74-0.88, and high convergent (correlation with SF-36) and discriminant validity (e.g. severity and infections). The Haem-A-QoL was linguistically validated in 42 different languages.

### Treatment satisfaction

The **Hemo-Sat**_**A **_[29] is the first haemophilia-specific treatment satisfaction questionnaire for adult patients with haemophilia and for parents of children with haemophilia (Hemo-Sat_p_), which has been developed in Italy. Items were generated on the basis of focus groups with haemophilia patients and an expert panel with haemophilia treaters and a pharmcoeconomist working in haemophilia. The Hemo-Sat_A _consists of 34 items pertaining to six dimensions ('ease and convenience', 'efficacy', 'burden', 'specialist', 'centre' and 'general satisfaction'). The questionnaire shows quite satisfactory psychometric characteristics in terms of reliability (Cronbach's α = 0.71-0.95) and validity (convergent: correlation with life satisfaction scale; discriminant: differences for clinical subgroups concerning severity and target joints) [30]. The Hemo-Sat_A _is available in 34 languages and linguistically validated in 24 languages.

### Other patient-rated outcomes (Functioning)

**HEP-Test-Q **is a newly developed questionnaire for the subjective assessment of physical functioning in adult haemophilia patients [32]. Its development was based on the training programme of the 'Haemophilia and Exercise Project (HEP)' [46]. Items were chosen together with experts in sports medicine and PROs development based on different aspects included in the modular training programme for haemophilia patients. HEP-Test-Q was tested in haemophilia patients in Germany and consisted of 25 items pertaining to four dimensions ('mobility', 'strength and coordination', 'endurance', 'body perception') and one additional item evaluating the physical activity compared with the last year to be analyzed separately. The psychometric characteristics showed good values for reliability (Cronbach's α = 0.85-0.96) and validity (criterion, convergent). The HEP-Test-Q is linguistically validated in German, English and Italian. Additional versions in Dutch, Greek, French and Spanish are available.

#### Linkage of the PRO measures to the ICF/ICF-CY

The contents of the five selected PRO measures were examined by extracting the meaningful concepts contained in the items of each measure and linking them to the ICF or ICF-CY [47]. The meaningful concept represents the first step in the linking process and it is represented by the extraction of the key meaning included in one item. For example, the item No.18 in the Medtap questionnaire: "I am afraid of internal bleeding" contains two different meaningful concepts "to be afraid" and "bleeding". In order to link items, established linking rules were adopted [43,47], which contain the following aspects: a) each item of a health-status measure should be linked to the most precise ICF category, b) if a single item encompasses different constructs, the information in each construct should be linked, c) the response options of an item are linked if they refer to additional constructs, d) if the information provided by the item is not sufficient for making a decision about which ICF category the item should be linked to, this item is assigned *nd *(not definable), e) if an item is not contained in the ICF classification, then this item is assigned *nc *(not covered by ICF).

The adult measures were linked by the ICF, while the only paediatric instrument (Haemo-QoL) was linked by the ICF-CY. Since the age-group version III of the Haemo-QoL contains the same items as the younger versions, but include more items and domains as the younger age-group versions, it has been decided to link only the oldest age-group version III; in this way the other younger age-group versions can be linked automatically in a subsequent step accordingly.

The linking process was carried out by 3 health professionals according to the description or definition of the item of the instrument in the literature. The number of concepts identified in each questionnaire and the ICF categories linked therewith were reported both in total and separated by component as shown in table 2.

**Table 2:** Percentage agreement between trainees.

	% Agreement		Chapter			
	**Number of concepts linked**	**component**	**1st level**	**2nd level**	**3rd level**	**4th level**

**Haemo-QoL**	**158**	77%	77%	76%	84%	

**Haem-A-QoL**	**92**	96%	89%	87%	79%	99%

**MedTap**	**78**	91%	87%	85%	87%	

**Hemo-Sat**_**A**_	**72**	92%	87%	87%	89%	

**HEP-Test-Q**	**40**	82%	75%	72%	82%	

**overall agreement**	**440**	**88%**	**83%**	**81%**	**84%**	

**Figure 2:**
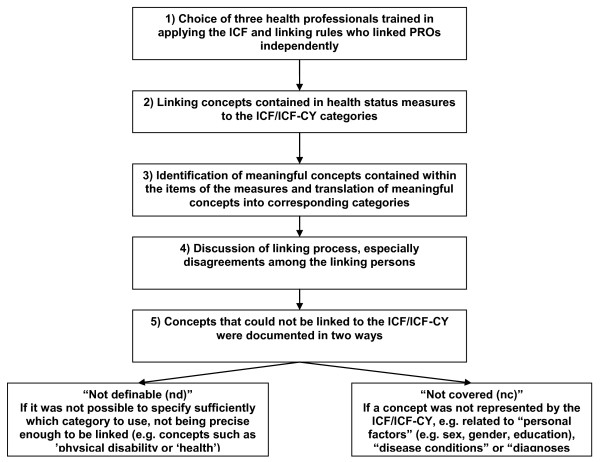
**Description of linking process**.

Agreement among 3 health professionals was used to determine which concepts were identified in all items of the questionnaires and which ICF category should be linked to each concept. In case of disagreement among the 3 health professionals, an expert in the field of ICF linking rules (Alarcos Cieza) was consulted. Pros and cons for the identification of different concepts and specific ICF category were discussed. On the grounds of these statements, the ICF expert took an informed decision. The linking procedure is described in figure 2.

### Inter-Rater Reliability

The reliability of the linking process was evaluated by calculating kappa coefficients [48] and 95% bootstrap confidence intervals [49] based on the two independent linking versions of each instrument. Kappa statistics were calculated per component, at the first, second, and third ICF level in order to indicate the level of agreement between the two health professionals conducting the linking procedure (see table 3).

**Table 3:** Kappa coefficients and non parametric bootstrapped 95%-confidence intervals for the linking procedure of the selected instrument for each questionnaire on the third level.

	Kappa coefficients	95% confidence interval
**Haemo-QoL**	**0.88**	0.80-0.97

**Haem-A-QoL**	**0.88**	0.86-0.95

**MedTap**	**0.88**	0.82-0.96

**Hemo-Sat**_**A**_	**0.85**	0.80-0.97

**HEP-Test-Q**	**0.85**	0.79-0.89

## Results

**Figure 3:**
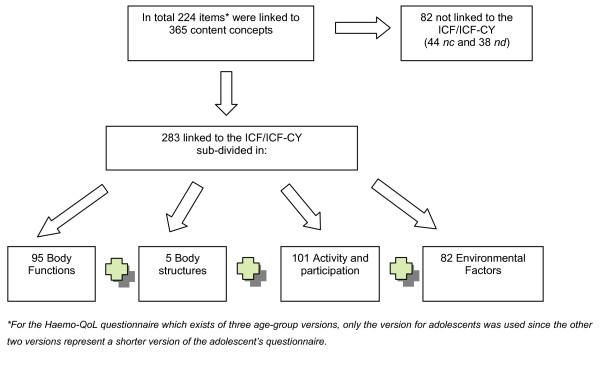
**Number of meaningful concepts identified in the 5 PROs and their distribution across the major components of the ICF/ICF-CY**.

Results will be presented according to meaningful concepts; ICF categories used for the linkage according to ICF component and for level of ICF hierarchy (see figure 3).

### Meaningful concepts

Figure 3 provides an overview of the number of the identified meaningful concepts and their distribution across the major components of the ICF/ICF-CY. Out of the 365 concepts identified, 283 could be linked to the ICF/ICF-CY (78%).

Most concepts addressed contents from the components "Activities and Participation" (36%) and 'Body Functions' (33%), followed by the component 'Environmental Factors' (29%). In contrast, only 2% of the concepts were linked to 'Body structures' (see figure 3).

Forty-four concepts out of 365 could not be linked to the ICF/ICF-CY and were coded *'not covered' (nc)*. These concepts were personal factors encompassing individual characteristics, such as self-perception, perception of others and perception of life (e.g. *"my haemophilia was a burden for me" (Haem-A-QoL)). *It is also important to underline the fact that thirty-eight out of the 365 concepts are *'not definable' *(*nd) *indicating that these concepts express rather general and unspecific concepts (e.g. *"I am well informed about hemophilia" (Haemo-QoL)*).

Out of the 70 different categories linked with the 5 PROs, 8 categories belong to the first level of hierarchy, 33 to the 2^nd ^level, 28 categories were linked on 3^rd ^level and 1 was represented on 4^th ^level (see table 4).

**Table 4:** The number of identified meaningful concepts in the selected haemophilia measures and the number of different ICF (ICF-CY) categories used for linkage distributed by ICF/ICF-CY components and level of hierarchy.

Concepts & Categories		Children		Adults			
				
	Total (Children/Adults)	Haemo-QoL	Total Adults	Haem-A-QoL	MedTap	**Hemo-Sat**_**A**_	HEP-Test-Q
**number of items**	**224**	**77**	**147**	**46**	**41**	**34**	**26**

**number of meaningful concepts (total)**	**365**	**123**	**242**	**83**	**62**	**62**	**35**

concepts linked to the ICF (Percentage)*	**283 (78%)**	**83 (67%)**	**200 (83%)**	**60 (72%)**	**51 (82%)**	**59 (95%)**	**30 (86%)**

concepts not linked to the ICF (total number)**	**82 (22%)**	**40 (33%)**	**42 (17%)**	**23 (28%)**	**11 (18%)**	**3 (5%)**	**5 (14%)**

**ICF/ICF-CY categories used for linkage (total number)**	**70**	**39**	**54**	**25**	**27**	**11**	**18**

**for level of ICF hierarchy**							

1st level	8	4	7	2	3	-	2

2nd level	33	22	21	10	11	7	6

3rd level	28	12	25	12	13	4	10

4th level	1	1	1	1	-	-	-

*Percentages are calculated based on the total number of concepts for each instrument;

**For ICF the total number of categories is 1,454 (Body functions: 493, Body structures: 310, Activity and Participation: 393, Environmental Factors: 258). For IC- CY the total number of categories is 1,685 (Body functions: 531, Body structures: 329, Activity and Participation: 552, Environmental Factors: 273) [35,39]

Tables 5, 6, 7, 8 show the coverage of ICF categories from the components 'Body Functions', 'Body Structure', 'Activities and Participation', and 'Environmental Factors' by the selected measures. None of the ICF categories was contained in all instruments. The most frequently used categories were:

**Table 5:** Frequencies of ICF/ICF-CY categories addressed in the different haemophilia-specific PROs in the component 'Body Functions'.

CODES	CHILDREN	ADULTS			
	**Haemo-QoL**	**Haem-A-QoL**	**MedTap**	**Hemo-Sat**_**A**_	**HEP-Test-Q**

**b1265** **Optimism**	3	2	2		
**b1266** **Confidence**			1		2
**b1300** **Energy Level**					1
**b140** **ATTENTION FUNCTIONS**		1			
**b1400** **Sustaining attention**	1*				
**b1401** **Shifting attention**		1			
**b152** **EMOTIONAL FUNCTIONS**	12	10	15	5	
**b1801** **Body image**	2	1			3

**b280** **SENSATION OF PAIN**	3	1	1		2
**b2801** **Pain in body part**		1			
**b28016** **Pain in joints**	1	2			

**b430** **HAEMATOLOGICAL SYSTEM FUNCTIONS**	1*				
**b4303** **Clotting functions**		3	2	4	
**b455** **EXERCISE TOLERANCE FUNCTIONS**					5
**b4551** **Aerobic capacity**					1

**b7** **Neuromusculoskeletal And Movement Related Functions**					2
**b710** **MOBILITY OF JOINT FUNCTIONS**	1		1		
**b7101** **Mobility of several joints**	1*				
**b730** **MUSCLE POWER FUNCTIONS**					1

**TOTAL**	**25**	**22**	**22**	**9**	**17**

* Category only included in the paediatric Haemo-QoL instrument

**Table 6:** Frequencies of ICF/ICF-CY categories addressed in the different haemophilia-specific PROs in the component 'Body structures'.

CODES	CHILDREN	ADULTS			
	**Haemo-QoL**	**Haem-A-QoL**	**MedTap**	**Hemo-Sat**_**A**_	**HEP-Test-Q**

**s730** **STRUCTURE OF UPPER EXTREMITY**	1*				
**s750** **STRUCTURE OF LOWER EXTREMITY**	1*				
**s770** **ADDITIONAL MUSCULOSKELETAL STRUCTURES RELATED TO MOVEMENT**	1*				
**s7701** **Joints**	1*				1

**TOTAL**	**4**	-	-	-	**1**

* Category only included in the paediatric Haemo-QoL instrument

**Table 7:** Frequencies of ICF/ICF-CY categories addressed in the different haemophilia-specific PROs in the component 'Activities and Participation'.

Codes	Children	Adults			
	**Haemo-QoL**	**Haem-A-QoL**	**MedTap**	**Hemo-Sat**_**A**_	**HEP-Test-Q**

**d161** **DIRECTING ATTENTION**	1*				

**d230** **CARRYING OUT DAILY ROUTINE**	1	1	2	2	

**d4** **MOBILITY**	1	1			
**d430** **LIFTING AND CARRYING OBJECTS**					1
**d4300** **Lifting**			1		
**d4302** **Carrying in the arms**					1
**d450** **WALKING**			1		1
**d4501** **Walking long distances**	1	1			1
**d4502** **Walking on different surfaces**					1
**d4551** **Climbing**			1		4

**d5** **SELF-CARE**		1			
**d570** **LOOKING AFTER ONE'S HEALTH**	1			1	
**d5702** **Maintaining one's health**	4	3		2	

**d6** **DOMESTIC LIFE**			2		

**d7** **INTERPERSONAL INTERACTIONS AND RELATIONSHIPS**	1*				
**d710** **BASIC INTERPERSONAL INTERACTIONS**	1*				
**d740** **FORMAL RELATIONSHIPS**	1			2	
**d750** **INFORMAL SOCIAL RELATIONSHIPS**					1
**d7500** **Informal relationships with friends**	4		1		
**d7504** **Informal relationships with peers**	1*				
**d760** **FAMILY RELATIONSHIPS**	1		1		
**d7600** **Parent-child relationships**		1			
**d770** **INTIMATE RELATIONSHIPS**	2	3	1		

**d820** **SCHOOL EDUCATION**	4	4	2	1	
**d830** **HIGHER EDUCATION**		4			
**d835** **SCHOOL LIFE AND RELATED ACTIVITIES**	3*				
**d8450** **Seeking employment**			1		
**d8451** **Maintaining a job**			1		
**d850** **REMUNERATIVE EMPLOYMENT**		4	2	1	
**d880** **ENGAGEMENT IN PLAY**	1*				

**d9** **COMMUNITY, SOCIAL AND CIVIC LIFE**					1
**d920** **RECREATION AND LEISURE**	1	2	2	2	
**d9201** **Sports**	4	3	1	3	1
**d9202** **Arts and culture**			1		
**d9205** **Socializing**			1		

**TOTAL**	**33**	**28**	**21**	**14**	**12**

* Category only included in the paediatric Haemo-QoL instrument

**Table 8:** Frequencies of ICF/ICF-CY categories addressed in the different haemophilia-specific PROs in the component Environmental Factors.

CODES	CHILDREN	ADULTS			
	**Haemo-QoL**	**Haem-A-QoL**	**MedTap**	**Hemo-Sat**_**A**_	**HEP-Test-Q**

**e1101** **Drugs**	3	5	3	23	
**e1201** **Assistive products and technology for personal indoor/outdoor mobility and transportation**		1			

**e3** **SUPPORT AND RELATIONSHIPS**	2		1		
**e320** **FRIENDS**	2*				
**e355** **HEALTH PROFESSIONALS**		2	1	7	

**e4** **ATTITUDES**	3		1		
**e410** **INDIVIDUAL ATTITUDES OF IMMEDIATE FAMILY MEMBERS**	5 *				
**e425** **INDIVIDUAL ATTITUDES OF ACQUAINTANCES, PEERS, COLLEAGUES, NEIGHBOURS AND COMMUNITY MEMBERS**	2 *				
**e430** **INDIVIDUAL ATTITUDES OF PEOPLE IN POSITIONS OF AUTHORITY**	1 *				
**e450** **INDIVIDUAL ATTITUDES OF HEALTH PROFESSIONALS**				2	
					
**e580** **HEALTH SERVICES, SYSTEMS AND POLICIES**				1	
**e5800** **Health services**	3	2	2	10	

**TOTAL**	**21**	**10**	**8**	**43**	-

* Category only included in the paediatric Haemo-QoL instrument

- "b152: Emotional functions'" which is contained in 42 items of the 4 different instruments

- "e1101: Products or substances for personal consumption", which is contained in 34 items of the 4 different instruments.

Only in HEP-TEST-Q the categories "b152" and "e1101" were not used.

### Representation of body functions

"Mental functions (b1)" were covered by all examined instruments. HRQOL instruments address more mental functions than other PRO questionnaires and are covered more in detail in the Haemo-QoL and the Haem-A-QoL than, for example, in the Hemo-Sat_A_. "Optimism (b1265)" which includes mental functions producing a personal disposition that is cheerful, buoyant and hopeful, is represented in all HRQoL instruments, but not in the other PROs. "Pain" is covered more in detail in the Haem-A-QoL than in the other PROs measures. "Clotting functions (b4303)", specific haematological system functions related with haemophilia, are represented in the Haem-A-QoL, Hemo-Sat_A _and the Medtap. Finally, "Energy level (b 1300)", mental functions producing vigour and stamina, is only presented in the HEP-Test-Q (see table 5).

### Representation of body structures

The component of 'Body Structure' is not well represented in all instruments. Only two instruments, the paediatric Haemo-QoL and the adult HEP-Test-Q address 'Body Structures', whereas the Haemo-QoL covers 'Body Structures' more in detail than the HEP-Test-Q. The Haemo-QoL includes four categories from the component of 'Body Structures'. Both instruments address aspects of structures related to movement (s7). "Structure of joints" is the only category included in both instruments (see table 6).

### Representation of activities and participation

"Carrying out daily routine (d 230)" is not contained in the HEP-Test-Q while it is covered by all the other PRO instruments. "Aspects of mobility (d4)" are well represented in the HEP-Test-Q and in the Medtap questionnaire, but they are scarcely represented in the Haemo-QoL, Haem-A-QoL and in Hemo-Sat_A_. "Self-care (d5)" is more broadly covered in Haemo-QoL, Haem-A-QoL and Hemo-Sat_A. _The chapter "Interpersonal interactions and relationships (d7)" is represented more in detail in the Haemo-QoL, the Medtap and in the Haem-A-QoL questionnaires. The concept of "school education (d820)" is especially covered by Haemo-QoL. With the exception of the Hemo-Sat_A_, "recreation and leisure (d920)" is addressed in the other PROs instruments. The Medtap covers in this category on the third level "sport (d9201)", "arts and culture (d9202)" and "socializing (d9205)" (see table 7).

### Representation of environmental factors

Four instruments, the Haemo-QoL, the Haem-A-QoL, the Medtap, and the Hemo-Sat_A _address environmental factors, whereas the Haemo-QoL covers environmental factors more in detail than the other three PRO instruments. The Hemo-Sat_A _has the highest frequency of categories from the component 'Environmental Factors'. The Haem-A-QoL covers two categories in the chapter "Products and Technologies", namely "Drugs (e1101)" and "Assistive products and technology for personal indoor and outdoor mobility and transportation (e1201)".

Within chapter 1, the ICF category "drugs (e1101)" is the most frequently used category. The category "drugs (e1101)" is addressed 23 times in the Hemo-Sat_A_. The Haemo-QoL particularly covers categories within the chapter "support and relationships (e3)" and the chapter "Attitudes (e4)", i.e. "the attitude of immediate (e410), peers and colleagues (e425) and the health professionals (e450)". The Medtap also contains "support and relationships (e3)" and "attitudes (e4)" at a general level (e3). Hemo-Sat_A _especially covers the category "Health professionals (e355)" from the chapter support and relationship. Finally, the category "Health services (e5800)" is represented in each of the four instruments which address environmental factors (see table 8).

### Comparison of Haemophilia measures for children and adults

One questionnaire for children (Haemo-QoL) and four instruments (Haem-A-QoL, Medtap, Hemo-Sat_A _and the HEP-Test-Q) for adult patients were selected. Out of the 70 ICF/ICF-CY categories identified for the linkage of the instruments' meaningful concepts, 17 (24%) categories were only addressed in the Haemo-QoL (categories with asterisk, Tables 5, 6, 7, 8) while the other 53 (76%) categories were addressed in the PRO measures for adults.

Within the 16 categories from the Haemo-QoL, three specific ICF-CY categories were used: "Focusing Attention (d161)", "School Life and related activities (d835)", "Engagement in play (d880)". They all belong to the component "Activities and Participation" (see table 7).

The subsequent categories differed between the related measures of the two age groups. The category "Emotional functions (b152)" had the highest frequency within the children's measure followed by "Individual attitudes of immediate family members (e410)" (n = 5), "Maintaining one's health (d5702)" (n = 4), "friends (d7500)" (n = 4) and "Sports (d9201)" (n = 4). The category "drugs (e1101)" had the highest frequency within the adult measures followed by "Health services (e5800)" (n = 14), "Health professionals (e355) "(n = 10), and "Clotting functions (b4303)" (n = 9) and "Remunerative employment (d850)" (n = 7).

Differences occurred within the component of "Environmental Factors (e)", which was more frequent in measures for adults, while the component "Body Structures (s)" was more used in the paediatric questionnaire. Regarding "Activities and Participation (d)", three new categories from the ICF-CY were used. Finally, concerning the ICF component "Body Functions (b)" no systematic differences were apparent between the paediatric and adult measures.

## Discussion

This study analyzed and compared the contents of 5 PRO questionnaires that can be used in haemophilia. The analysis was based on the ICF and the ICF-CY and provided relevant information on the contents of these instruments. The results can guide researchers in selecting and reporting haemophilia questionnaire outcomes in clinical trials and observational studies in which HRQoL and other PROs are among the endpoints. Moreover, this analysis can help choosing a questionnaire for rehabilitation professionals in their clinical practice. Specifically, selection could be made depending on aspects of haemophilia, which are included in the different instrument (using the ICF/ICF-CY as the basis), that are relevant for the aim of a particular study [46]. This study does not aim to propose which of the questionnaires are preferred. The questionnaires have been developed for different purposes and their focus to haemophilia varies, reflecting the complexity of this disease and its management.

In general, HRQoL measures represented more categories in 'body functions', 'body structures' and 'activities and participation', while assessments of physical functioning such as the HEP-Hest-Q represented more categories in 'environmental factors'. Researchers and clinicians can identify the components they want to measure. A researcher interested in physical endurance can examine the category "Exercise tolerance functions (b 455)" under 'body functions' and see that this category is represented in the HEP-Test-Q and may therefore present an adequate and more precise choice.

Not only did instruments differ in precision, but also in the level of differentiation regarding the level of activity and proficiency [50], an aspect which is important to cover for the whole spectrum of activities encountered in a heterogeneous number of adult populations - young or old adults, sedentary or sporting, healthy or ill people. An example is the HEP-Test-Q, which covers "walking", "walking long distances", "walking on different surfaces", and "climbing".

Concerning 'body structures' only two measures represented categories related to movement. However, in some clinical settings (e.g. prophylaxis for children) or in the context of rehabilitation (e.g. orthopaedic rehabilitation) instruments representing 'body structures' such as Haemo-QoL and HEP-Test-Q are preferable.

### Adults and children

With regard to content representation, differences between measures for adults and children appeared. Haemo-QoL more often addressed 'Body Structures' and 'Activities and Participation' components than adults' measures. In contrast, 'Environmental Factors' were more often addressed in the selected instruments for adults.

"Emotional functions (b152)" were mainly found in children's measure, which can be explained by the stronger emotional impact of a chronic disease on children, while "drugs (e1101)" were mainly represented within adult measures, demonstrating the predominant burden related to the dependence from medication in adult patients.

Considering the most frequently used ICF categories, further differences between the contents of Haemo-QoL and the adult-specific instruments were found. Within the Haemo-QoL, "friends", "school", "recreation and leisure" and "family relations" were addressed most often. These are the areas where the most difficult problems may arise in relation to children with a chronic health condition like haemophilia [51].

Most frequently addressed areas within the adult-specific measures were "drugs and treatment", "work", "sport" and "bleeding" representing the direct impact of haemophilia on patients' lives [27].

Although the results confirm that ICF and ICF-CY provide a good framework for content comparisons of PRO instruments, several limitations apply to this study. We conducted a systematic literature review to select current condition-specific PRO measures applied in haemophilia choosing the most frequent measures used in Europe but this literature review is not comprehensive on a worldwide level. Furthermore, we did not have access to all published haemophilia-specific instruments such as the Hemofilia-QoL, why only those instruments were included in the linking process, which were accessible. The Hemophilia Activity List (HAL) [31] was already linked by the authors, why we didn't repeat the linking process for this instrument.

The ICF/ICF-CY was not specifically designed for describing drugs, resulting in the fact that several concepts related to the use of treatment (especially in the Hemo-Sat_A _questionnaire) were not well represented in the ICF/ICF-CY. Moreover, all the concepts referring to "personal factors" identified in the instruments could not be linked since they are not yet contained in the current ICF and ICF-CY versions. If several meaningful concepts are not covered by the ICF, the risk to have a considerable loss of information from questionnaires can increase. Problems are related also with the linking procedure. The linking process revealed that the category emotional functions (b152) were very frequently addressed in the different instruments, which may indicate that the ICF/ICF-CY does not sufficiently differentiate feelings and emotions.

On the other hand, the linking process as a method does not always consider the contextualization that is included in one item. For example, in the Haemo-QoL item "it was difficult for me to move my arms or legs", the linking procedure linked three concepts, "b710 mobility of joint functions", "s730 structure of upper extremity", "s750 structure of lower extremity". However, it can be stated that "moving limbs" in this sense contextualizes all the activities related with the upper extremities and the lower extremities and this contextualization is not captured by the linking procedure.

In addition, the linking procedure does not consider time recall included in the item nor does it consider the directionality of cause and effect, if any. The directionality of concepts within one item is lost after identification of relevant ICF categories. For example, the item "injections annoyed me" from the Haemo-QoL refers to the adverse influence of the way to administer the treatment on patient's mood and feelings. In contrast, the item "I had problems walking downstairs" from the HEP-Test-Q asks about the impact of the disease for a specific action of walking.

From a methodological point of view, while the quality of linking was assured, the identification of meaningful concepts and coding based on the ICF not only requires of the linkers an in depth understanding of the ICF but also experience in linking. Hence the degree of dissent between linkers should always be considered. Finally, in the present paper, one disease-specific PRO for children and four PROs for adults were examined according to the inclusion criteria (availability in more than 2 European languages, self-report and accessibility). In the future, further PRO haemophilia measures for children should be linked in order to broaden the choice, such as the Canadian CHO-KLAT [22].

In conclusion, the results confirm that, despite the evident limitations, ICF and ICF-CY provide a good framework for content comparisons of PRO instruments, evaluating similarities and differences in respect of ICF/ICF-CY concepts. Since the ICF classification is the basis of the linking process and provides a common language for clinical practice, teaching and research, it will probably become the essential reference for existing PRO measures, as well as for PRO measures to be developed in the future [52].

Because of their linkage with ICF/ICF-CY, haemophilia-specific PROs can be used to estimate the prevalence of disability and health in specific patient populations. For example, the ESCHQoL study provides PRO data from more than 1,400 haemophilia children and adults from 21 European countries [53], which can be used to calculate the prevalence of functional impairment and consequently the burden of this disease.

Finally, ICF and ICF-CY appear to be valuable tools in practical work, for clinical diagnosis, for planning intervention and for facilitating communication among professionals.

## Competing interests

The authors declare that they have no competing interests.

## Authors' contributions

RS, as first author, carried out the linking procedure, performed the statistical analysis, participated in the design of the study and drafted the manuscript. BM was principal investigator, developed the design of the study and participated in writing of the paper. AE participated in the linking procedure. vMS participated in the development of the design and the coordination the study, carried out the linking procedure and participated in the analysis of the data and writing of the paper. All authors read and approved the final manuscript.

## Ackowledgements

The first author was a Marie Curie Fellow and acknowledges support from the EC project with the contract number MRTN-CT-2006-035794 (MURINET) - MARIE CURIE ACTIONS, Marie Curie Research Training Networks (RTN) for the opportunity of conducting this study and for the provision of the different haemophilia-specific questionnaire for the linking process.
